# Beyond Single Variants: A Pathway-Based Approach to Explore the Genetic Basis of Memory

**DOI:** 10.1007/s12035-025-05582-1

**Published:** 2026-01-15

**Authors:** Daan van Beek, Martina Kutmon, Theo de Kok, Ilja Arts, Michelle Moerel, Michiel Adriaens

**Affiliations:** 1 Maastricht Centre for Systems Biology and Bioinformatics (MaCSBio), Maastricht University, 6229 EN Maastricht, Netherlands; 2 Department of Translational Genomics, GROW School for Oncology and Developmental Biology, Maastricht University Medical Centre, 6229 ER Maastricht, Netherlands; 3 Department of Cognitive Neuroscience, Faculty of Psychology and Neuroscience, Maastricht University, 6229 ER Maastricht, Netherlands

**Keywords:** Human Connectome Project, Memory, Episodic memory, GWAS, Pathway analysis, Network biology

## Abstract

Memory plays a crucial role in human cognitive processes and daily functioning. While memory has a genetic basis, identifying the specific genetic factors influencing memory performance has proven challenging. This challenge arises because memory is a complex trait, whose genetic architecture likely comprises an accumulation of many low-effect size common variants. Thus, study sample sizes are easily insufficient, leading to underpowered genetic association analyses. This limitation is especially pronounced in studies that prioritize deep phenotyping over broader recruitment, resulting in smaller cohorts. Given these limitations, important biological signals may remain undetected when relying solely on conventional genome-wide significance thresholds. However, even variants that fall below these thresholds can yield meaningful insights when analyzed in a broader biological context. Therefore, we propose that relevant biological information can still be extracted by employing a less stringent *p*-value threshold paired with elaborate variant mapping and functional annotation to identify candidate variants, genes, and biological pathways associated with memory performance. We present three independent genome-wide association studies within the Human Connectome Project on a (i) Penn-Word verbal episodic memory test (*n* = 1131), a (ii) Picture Sequence visual episodic memory test (*n* = 1133), and a (iii) List Sorting verbal working memory test (*n* = 1134). Subsequent variant mapping, functional annotation, and pathway identification were performed using FUMA, Cytoscape, KEGG, Reactome, and WikiPathways. At the pathway level, but not single variant or gene level, we observed substantial overlap in results across the three memory tests, and between our results and previously reported findings. Several identified genes and pathways were previously associated with memory-related disorders, and processes related to cognition, neurodevelopment, and neurological dysfunction. We interpret the common pathways as reflecting shared biological mechanisms underlying memory. Our findings underscore the potential of our proposed approach, which we provide as an openly accessible pipeline, for exploring other complex polygenic traits.

## Introduction

A pivotal aspect of cognition and behavior is the brain’s capacity to encode, store, and recall information about the outside world as memories. The importance of this capacity becomes evident in Alzheimer’s disease, which affect a person’s ability to efficiently navigate and interpret the world and can even lead to the erosion of individual history and identity [33]. Memory impairments are also observed across a range of psychiatric conditions. For example, depression and schizophrenia are frequently associated with impaired working memory performance [14, 17, 21]. Recognizing the societal impact of memory impairment, extensive research has focused on uncovering the biological mechanisms underlying memory [37]. Central to memory formation, consolidation, and retrieval is the concept of neuroplasticity, which is the brain’s ability to adapt and reorganize in response to internal or external stimulation [3]. At a larger spatial scale, neuroplasticity involves alterations in the connectivity within and between brain regions. Large-scale changes in brain connectivity are driven by mechanisms of neuroplasticity that act at a smaller spatial scale. Such mechanisms include, for example, modifications of dendritic tree size and spines, axonal myelination, and synapse number, but also the formation of new neurons [28]. Importantly, these processes operate through biological pathways that are genetically regulated [27].

Accordingly, evidence from population studies suggests that genetic factors play a substantial role in memory capabilities. It is estimated that genetic differences explain 30–60% of the variability in individuals’ memory task performance, suggesting a moderate to high heritability. In pursuit of understanding these genetic influences, genome-wide association studies (GWAS) have been employed to identify genetic markers associated with memory performance (*n*_min_ = 341; *n*_max_ = 162.335) [9, 26, 42, 43, 54, 60, 74]. However, these efforts have faced significant obstacles. A major challenge has been the difficulty in replicating results across studies, and consequently, there is no consensus on the genes underlying memory [56].

Cognitive traits like memory present several significant challenges for genetic research. First, memory is a complex trait, likely influenced by thousands of genetic variants each with a low effect size [39, 73]. Consequently, studies into the genetics of memory are easily underpowered, which is most likely contributing to discrepancies in results across previous studies. Second, memory is not a single entity but rather a multifaceted construct. While the biological underpinnings of different memory types may partially overlap, they also likely diverge in key aspects. The primary division in memory types is that between working memory (WM) and long-term memory, with working memory operating over much shorter timescales than long-term memory [2]. Episodic memory (EM), which enables the recollection of personal experiences and specific events, is a key example of long-term memory [58]. While long-term memory formation depends on gene expression that strengthens existing neural connections and forms new ones, working memory relies on existing neural networks across the brain [2, 8]. Previous GWAS studies of memory, both in healthy and diseased populations, often differed in the type of memory studied [9, 26, 54, 74]. Even within one memory type, there are different test types focusing on either numerical, visual, or verbal memory [63]. Heterogeneity in memory constructs and tests likely contributes to inconsistent genetic findings across studies. Finally, studies focusing on complex phenotypes often prioritize deep phenotyping, enabling integration of to derive mechanistic insights. However, due to budget and time constraints this often results in smaller cohorts, reducing the statistical power that is needed to detect genome-wide significant single-nucleotide polymorphisms (SNPs).

Given these challenges, studies into the genetics of memory may overlook important biological signals when relying only on conventional genome-wide significance thresholds. That is, sub-threshold variants can offer valuable insights, especially when considered collectively within a biological context. Therefore, we propose the use of a lenient *p*-value threshold paired with extensive variant mapping, functional annotation, and pathway identification. In contrast with a traditional GWAS, which tests the effect of individual genetic variants, the applied functional analysis based on pathway information can identify which groups of closely related genes are associated with the trait. This allows for the joint biological interpretation of genetic variants below the genome-wide significance threshold, which would otherwise not be considered [64]. This approach is therefore hypothesized to be especially advantageous when analyzing data from underpowered cohorts, where a GWAS likely fails to detect significant associations [6, 25]. As this approach goes beyond single variants or genes, and instead examines biological mechanisms, we anticipate that investigating memory at the pathway level has the potential to reveal biological connections among GWAS findings that might initially appear unrelated. That is, assessment of the pathways that are shared across memory tests may allow moving towards a mechanistic understanding of the genetic commonalities across memory types.

Here, we therefore explore the genetic basis of memory using the Human Connectome Project (HCP) dataset [59, 65], which was selected because it reflects a representative use case for our proposed approach. The HCP is characterized by deep phenotyping across a range of cognitive and neural measures, including multiple memory tests. Consequently, the dataset has a relatively small sample size. This trade-off makes it an ideal testing ground for our proposed approach. Crucially, the HCP includes three distinct memory assessments, enabling analysis of both shared and unique genetic influences across memory subtypes. Moreover, the HCP contains multi-modal imaging (i.e., magnetic resonance imaging and magnetoencephalography) data, and thereby offers high potential for follow-up work if reliable genetic results can be obtained. We present the results of three separate memory test GWASs using this proposed approach to study the genetics of working memory (WM), verbal episodic memory (EM), and visual EM. We show that our approach results in biological pathways that are shared across memory types. While the individual methods we apply are well-established, our contribution lies in demonstrating their combined utility within the context of complex, polygenic traits and richly phenotyped, but statistically limited, datasets.

## Methodology

All scripts used in this pipeline, including annotation, can be found on GitHub (https://github.com/macsbio/HCP_Memory_GWAS).

### Data Acquisition

We analyzed behavioral and genetic data from the Human Connectome Project (HCP) Young Adult data release, consisting of 1,206 healthy North American individuals aged 22–35 years (Table 1) [65]. These participants came from 300 ethnically diverse families, including both twin and non-twin sibling pairs. From the available behavioral data, we focused on three memory tests. The Penn-Word Memory Test and Picture Sequence Test both assess episodic memory, respectively verbal and visual EM. Additionally, the List Sorting Test was included as a measure of verbal WM performance [65]. The Penn-Word Memory test is aimed at recalling 20 words from a list of those words mixed with 20 foil words [23]. In the Picture Sequencing test, participants need to order a set of pictured objects and activities that were presented with verbal description [13]. The List Sorting test requires participants to sort and sequence object illustrations, presented with a verbal description, based on given characteristics [57]. The genotyping was performed with a combination of the Illumina Neuro Consortium, (472,278 SNPs) and the ImmunoArray PsychArray (2,119,803 SNPs). Genotypes were imputed by the HCP following the Marchini 1000 Genomes approach. Details on data collection can be found in the HCP Young Adult 1,200 Subjects Data Release Manual [24].

**Table 1:** Descriptive statistics of the 1140 genotyped individuals in the study

Age	Minimum = 22; median = 29; maximum = 36
Sex	522 males; 618 females
Ethnicity	844 white; 178 black or African American; 64 Hawaiian or Other Pacific Islander; 29 More than one; 23 Unknown or Not Reported; 2 American Indian or Alaskan Native or Asian Native

### Genome-Wide Association Study

Before conducting a separate genome-wide association study on each of the three memory tests, we pre-processed the genetic dataset as follows. Quality control was performed using the following acceptance criteria: minor allele frequency > 0.05, genotype call rate > 0.95, individual call rate.

 > 0.95, and Hardy–Weinberg equilibrium *p*-value > 1E^−6^. Kinship and ethnicity were assessed and adjusted for with the first 20 genomic principal components and a sparse genetic relationship matrix, derived from a full relationship matrix with a cut-off of 0.05. The principal component analysis was performed using PLINK and the genetic relationship matrix was computed using GCTA [47, 69]. Subsequently, a separate genome-wide association analysis was performed for each of the three memory tests following the GCTA MLMA-LOCO protocol, corrected for age and sex [70]. Heritability for Penn-Word and List Sorting was assessed using the GREML-LDMS approach [67]. For Picture Sequence, the REML procedure was used to assess heritability because too many variance components were constrained for GREML [68]. Quantile–quantile (QQ) plots and corresponding lambda values for each GWAS were generated in R 4.3.2 to assess potential p-value inflation [11, 48]. We performed GCTA Bivariate restricted maximum likelihood (REML) analysis to estimate genetic correlations between the different memory types [35]. Additionally, we assessed per-SNP effect size correlation between the GWAS results of each pair of memory tests.

### Variant Mapping and Functional Annotation

The summary statistics, consisting of the location, *p*-value, and variant effect size for each SNP, were then used for further analysis using FUMA, a platform designed for variant mapping and functional annotation [62]. We used the most lenient cut-off value to accept SNPs (*p*-value < 1E^−5^) in order to maximize the scope of the functional analysis. Lower *p*-values thresholds of 1E^−6^ have been suggested for exome-wide studies with MAF > 5% in the European Journal of Human Genetics [16]. In addition, the 1E^−5^ threshold has been used as a “suggestive” threshold in a New England Journal of Medicine GWAS on severe COVID-19 (Group, 2020) [20]. Given the ethnically diverse American study population, the 1000G Phase 3 ALL reference population was chosen. Expression quantitative trait locus mapping (eQTL) was performed in FUMA to identify genes whose expression is influenced by the variants with *p*-value < 1E^−5^. In FUMA, every SNP-gene eQTL pair is selected based on the predetermined *q*-value < 0.05 from each database. This analysis used all available brain tissue databases, including BRAINEAC, CommonMind Consortium, GTEx v8 Brain, and PsychENCODE [1, 7, 18, 55] The remaining parameters were set to default. Each resulting list of SNP-gene annotations was then filtered to include only those located in intronic and exonic regions of both coding and non-coding RNAs, 3’ and 5’ untranslated regions, and associations through expression quantitative trait loci. This resulted in lists of genes associated with memory test performances through the location and effect of the variants of interest for each GWAS. In addition, through FUMA, a MAGMA gene-based analysis of the GWAS results was performed [10].

### Pathway Identification and Network Visualization

Next, we analyzed the lists of candidate genes at the pathway level. We used the Reactome, KEGG, and WikiPathways databases to identify all pathways associated with at least one candidate gene, which produced a two-column table listing every gene-pathway association [15, 29, 44]. This table was imported into Cytoscape (version 3.9.1) using the RCy3 package to visualize the associations as a gene-pathway network [24, 52]. We created separate networks for each memory test (i.e., the Penn-Word, Picture Sequence, and List Sorting tests) and also merged the three separate networks to explore potential connections across the memory types. Furthermore, to show connections between genes through shared SNPs, we extended these networks to include the SNPs from the GWAS, LD analysis, and eQTL mapping. Finally, to account for pathway redundancy across the three pathway databases, the gene overlap between pathways was calculated and visualized to find groups of pathways with similar biological roles. Pathways with ≥ 80% shared genes were grouped together.

### Comparison to Similar Studies in the GWAS Catalog and Pathway Permutation

In order to confirm our findings on the HCP cohort, we performed the same FUMA and pathway identification pipeline on four studies across three cohorts with memory scores and genetic data available. Specifically, we extracted the GWA summary statistics of four studies on visual memory performance [9], numeric WM [26], visual prospective memory [26], and verbal EM [54] from the GWAS Catalog [53]. We have performed 100,000 permutations of Jaccard index pathway overlap with randomly drawn gene lists of the same size as our FUMA genes present in Reactome, KEGG, and WikiPathways. The results of these permutations are shown in supplementary figure F6.

## Results

We performed separate GWASs on the Penn-Word, Picture Sequence, and List Sorting memory tests within the Human Connectome Project cohort. The identified genetic variants with a lenient significance threshold (*p*-value < 1E^−5^) were imported into FUMA for functional mapping and annotation to create candidate gene lists. Finally, pathway associations for each candidate gene list were collected and the information was visualized in networks to facilitate biological interpretation.

### Data Pre-Processing

Out of the *n* = 1206 total individuals, *n* = 1131 samples had complete data for the Penn-Word, *n* = 1133 for the Picture Sequence, and *n* = 1134 for the List Sorting test. In total, 6,143,592 autosomal SNPs were accepted for genome-wide analysis based on minor allele frequency > 0.05, genotype call rate > 0.95, individual call rate > 0.95, and Hardy–Weinberg equilibrium *p*-value > 1E^−6^.

Statistical analysis revealed low but significant correlations between scores on the three memory tests (Table 2). Overall, the explained variances between the different memory tests were low (highest *R*^2^ = 0.09), suggesting that these tests measure at least partially non-overlapping cognitive traits.

**Table 2:** Correlation between memory test scores

*Test*	*Penn-Word (EM)*			*Picture Sequence (EM)*		
	*R*	*p-value*	*R*^*2*^	*R*	*p-value*	*R*^*2*^
*Picture Sequence (EM)*	0.22	< 2.2E^−16^	0.05			
*List Sorting (WM)*	0.16	< 2.2E^−16^	0.02	0.29	< 2.2E^−16^	0.09

Pearson Correlations (*R*-values), corresponding *p*-values, and explained variance (*R*^2^-values) between scores on the three memory tests. *EM*, episodic memory; *WM*, working memory

### The Primary GWAS Results

**Fig. 1:**
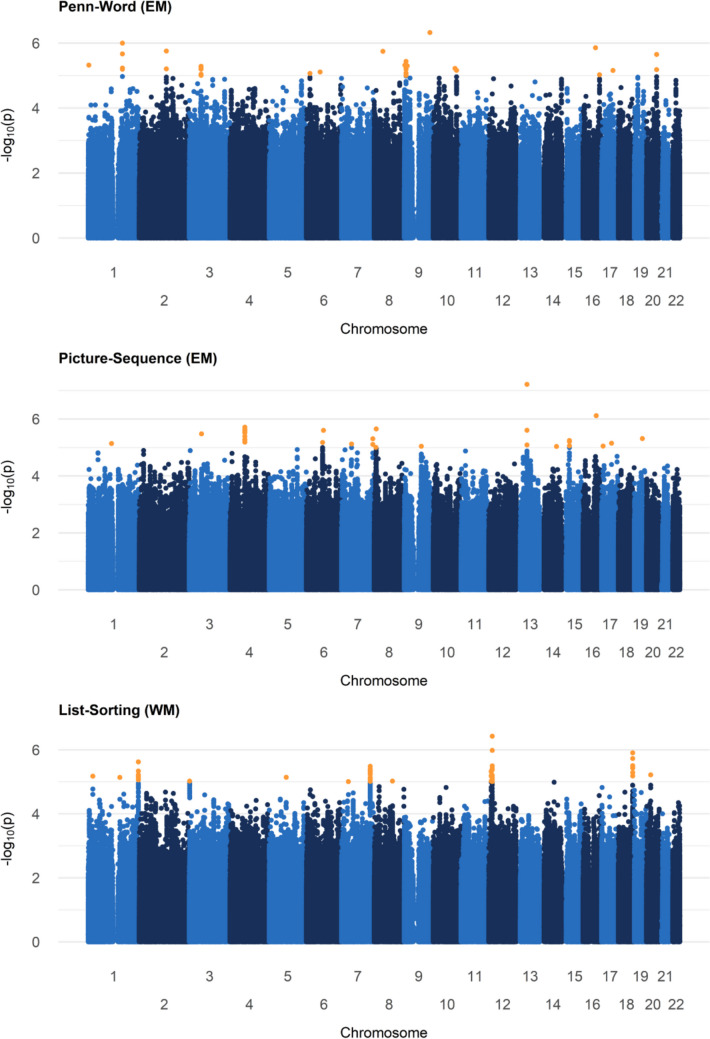
Manhattan plots for each of the performed GWASs. Orange dots highlight the SNPs with *p*-value < 1E^−5^

We found 17, 16, and 11 SNPs surpassing a lenient cut-off (*p*-value < 1E^−5^) for the Penn-Word, Picture Sequence, and List Sorting tests, respectively (Fig. 1, Table 2, and supplementary table T1, T2, T3). No variants reached genome-wide significance (*p*-value < 5E^−8^). In addition to the genome-wide associations, heritability estimates were computed. The heritability estimates for the EM tests were 39.1% and 42.0% for the Penn-Word Test and the Picture Sequence Test respectively. The List Sorting Test, assessing WM, had an estimated heritability of 61.7%. The lambda values of respectively 1.04, 1.11, and 1.13 for Penn-Word, Picture Sequence, and List Sorting were all close to 1, suggesting no *p*-value inflation or deflation. The corresponding QQ plots support this conclusion (supplementary figure F1). The results of each bivariate REML analysis, estimating the genetic correlation between the traits measured by the three memory tests, were highest between the Picture Sequence and Penn Word tests (rG = 0.59), followed by the Picture Sequence and List Sorting tests (rG = 0.56), and finally the Penn-Word and List Sorting tests (rG = 0.35). These results suggest a moderate shared genomic explained variance. For the top SNPs, those reaching p-value < 1E^−5^, there was no overlap between the three GWASs (supplementary table T4). There were significant correlations (*p* < 2.2E^−16^ for all) between the genome-wide per-SNP effect sizes of Penn-Word and Picture Sequence (*R* = 0.23), Penn-Word and List Sorting (*R* = 0.17), and Picture Sequence and List Sorting (*R* = 0.31) (supplementary figure F2).

### Functional Annotation

The SNPs resulting from each GWAS were then used for linkage disequilibrium (LD) analysis (*R*^2^ ≥ 0.6), which provided an expanded SNP set to be used for functional annotation. This resulted in respectively 25, 35, and 10 genes for the Penn-Word, Picture Sequence, and List Sorting test (Table 3, see supplementary table T5 for a list of all genes). For Picture Sequence, the *SERPINE3* gene reached a MAGMA *p*-value of 1.31E^−6^, close to the MAGMA genome-wide significance of *p*-value < 2.7E^−6^. There was no overlap between the genes resulting from each of the three analyses.

**Table 3:** Correlation between memory test scores

*Test*	*SNPs p-value* < *1E*^*−5*^	*SNPs r*^*2*^ > *0.6*	*Genes*
*Penn-Word (EM)*	17	315	25
*Picture Sequence (EM)*	16	359	35
*List Sorting (WM)*	11	263	10

The columns show the number of SNPs resulting from the GWAS, the number of SNPs resulting from LD association to be used for functional annotation, and the number of genes resulting from functional annotation of the LD-expanded SNP set.

### Pathway Identification

**Fig. 2:**
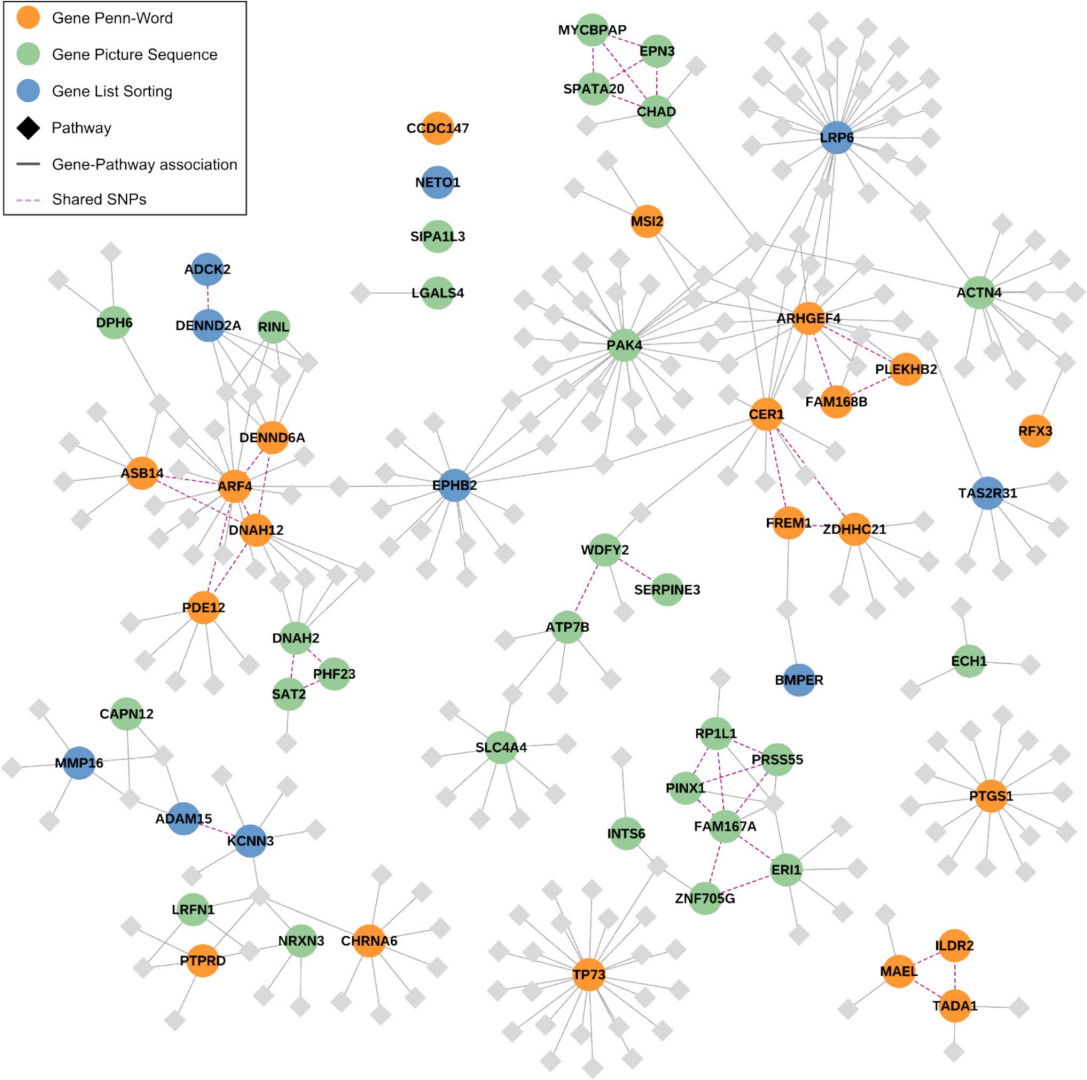
Gene-pathway network of functional annotation results. Network visualizing the results from the functional annotation and mapping visualization in Cytoscape, merged across all three memory tests (NDEx). Genes are presented in orange (Penn-Word), green (Picture Sequence), and blue (List Sorting). Pathways are shown as light gray squares. SNPs were excluded for clarity, and shared SNP connections between genes were replaced by direct dashed connections

We further explored the resulting lists of genes by using the Reactome, KEGG, and WikiPathways databases to find pathways in which these genes are present. We visualized the results for each memory test as networks in which genes were connected to (i) other genes if they have shared SNPs and to (ii) pathway nodes if they had a role in that pathway (supplementary figures F3, F4, and F5). The three networks were merged into one network to connect findings from the three different tests on a pathway level (Fig. 2). Interactive versions of all networks including node information were uploaded to NDEx (https://www.ndexbio.org) [45].

We identified 116 pathway associations from the Penn-Word test, 88 from the Picture Sequence test, and 66 from the List Sorting test, totaling 229 unique pathway associations. Pathway sizes ranged from 5 to 1463 with a median of 88.5. Among these, 38 pathways connected two or more genes, and 34 pathways connected genes across the different memory tests. Overall, genes from each memory test tended to cluster together. This clustering was due to shared SNP associations, for example around *SPATA20* (Fig. 2, green nodes, top) and *RP1L1* (Fig. 2, green nodes, bottom). Additionally, part of the memory test-specific clusters were driven by functional relations. For example, *MSI2* (Fig. 2, orange node, center top) is connected with its neighbors through Rho GTPase signaling.

### Description of the Merged Pathway Identifications

**Fig. 3:**
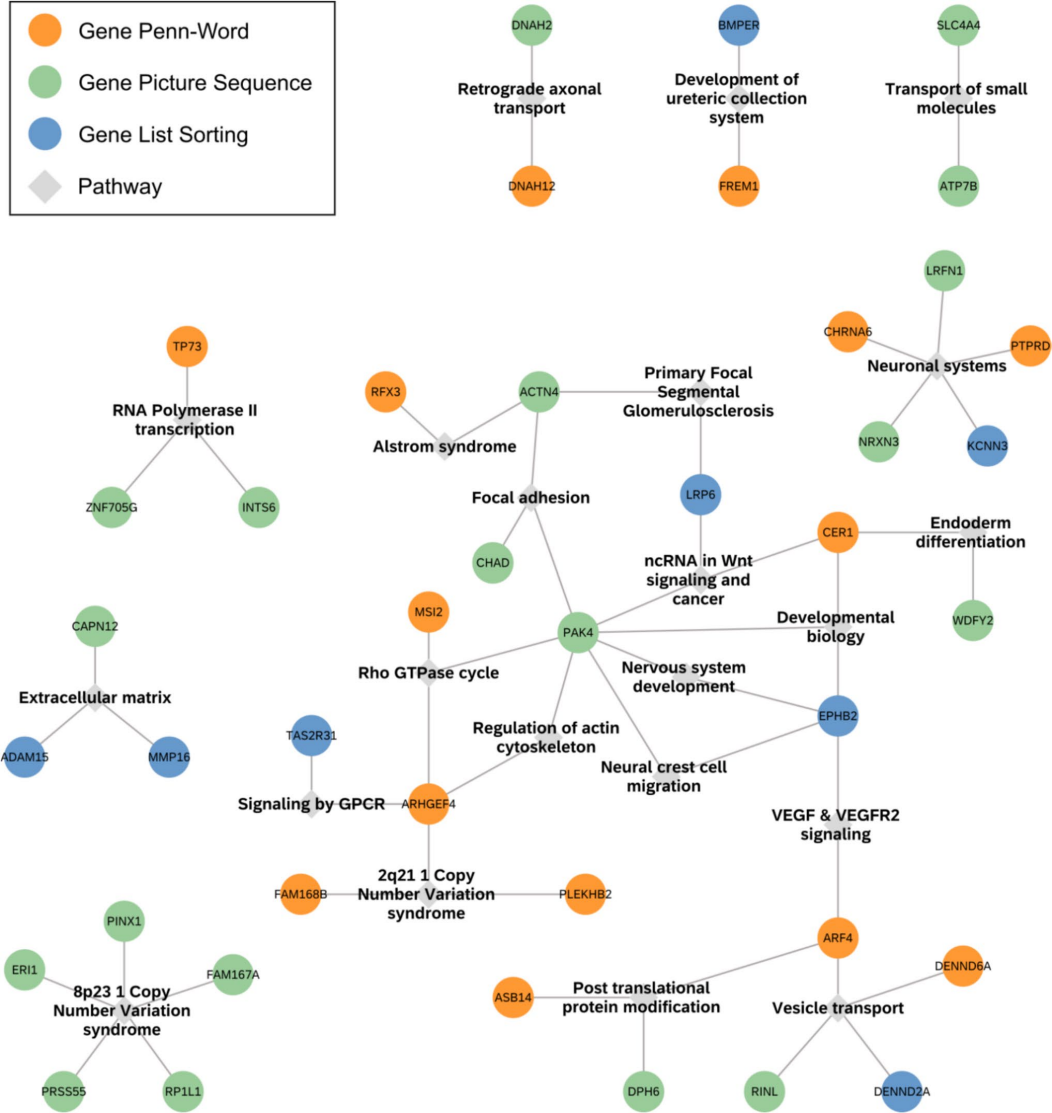
Shared pathways between the three gene lists. This network shows pathway connections between genes from the Penn-Word, Picture Sequence, and List Sorting results (NDEx). Pathways with ≥ 80% gene overlap were grouped together. Genes are presented in orange (Penn-Word), green (Picture Sequence), and blue (List Sorting). Light gray squares represent pathways. Full pathway names and group members are presented in supplementary table T6. Note that genes without shared pathway connections were excluded from this figure

While there was no overlap between the genes associated with the different memory tests, our pipeline revealed connections through shared pathways. We identified 38 shared pathway associations. After merging pathways with ≥ 80% shared genes for visualization and simplification purposes, 25 pathway nodes remained (Fig. 3, supplementary table T6). The 2q11.2 and 8p23.1 copy number variation pathways (bottom left of Fig. 3) connected several genes based on their location in the affected genomic regions. The pathway node with the most connections between the different gene lists was the node representing neuronal systems, which includes vesicle mediated transport, membrane trafficking, and protein–protein interactions at synapses.

### Comparison to GWAS Catalog Studies and Pathway Overlap Permutation

Our gene lists showed only limited overlap with those derived from performing the functional annotation pipeline on the summary statistics of four available GWAS Catalog studies (supplementary table T7). Specifically, two genes from the Picture Sequence analysis were also detected in the Hatoum et al. prospective memory analysis (referred to as dataset B in Table 4). The overlap at the gene level was similarly low among the four GWAS Catalog studies (Table 5 and supplementary table T7). Interestingly, at the pathway level, there was considerable overlap among the four GWAS Catalog studies and between these studies and our results (Table 5 and supplementary table T7), ranging up to an overlap as high as 68% between the Picture Sequence and the Hatoum et al. digit span analyses (referred to as dataset B in Table 5). There was similar pathway overlap between the two episodic memory tests within our cohort and between each of these with the working memory test. The Jaccard index pathway overlap from the three memory tests in our analyses represented the 61 st, 77th, and 84th percentile for randomly drawn gene lists of the same sizes for Penn Word versus Picture Sequence, Penn Word versus List Sorting, and Picture Sequence versus List Sorting, respectively (supplementary figure F6).

**Table 4:** Overlap quantified as gene count

		*HCP*			*Catalog*			
		*Penn-Word*	*Picture Sequence*	*List Sorting*	*A*	*B*	*C*	*D*
*HCP*	*Penn-Word*		0	0	2	0	0	0
	*Picture Sequence*			0	1	0	0	0
	*List Sorting*				1	0	0	0
*Catalog*	*A*					0	1	0
	*B*						2	0
	*C*							0
	*D*

**Table 5:** Overlap quantified as pathway percentage

		*HCP*			*Catalog*			
		*Penn-Word*	*Picture Sequence*	*List Sorting*	*A*	*B*	*C*	*D*
*HCP*	*Penn-Word*		19	17	46	65	26	12
	*Picture Sequence*	25		15	52	73	30	25
	*List Sorting*	20	20		62	55	15	21
*Catalog*	*A*	11	10	9		59	20	12
	*B*	7	6	3	25		11	10
	*C*	16	14	5	49	60		19
	*D*	8	12	8	31	63	20	

Overlap of results in terms of gene count (Table 4) and pathway percentage (Table 5, percentage of “row” shared with “column”) across datasets, which comprises the three HCP GWASs of the current study and four datasets retrieved from the GWAS Catalog. The GWAS Catalog datasets are referred to as follows: [A] Davies et al. (6), [B] Hatoum et al. digit span (5), [C] Hatoum et al. prospective memory (5), and [D] Soo et al. (7)

## Discussion

Investigating the genetic basis of memory must deal with the fact that memory is a multi-faceted, complex trait [39, 73]. Integrating different layers of biological and social data requires deep phenotyping, often at the cost of larger cohorts. We hypothesized that conducting a GWAS with a lenient *p*-value threshold followed by extensive biological interpretation can offer an effective approach for assessing the genetics of memory at the mechanistic level.

To test our proposed approach, we conducted three GWASs within the Human Connectome Project, focusing on the Penn-Word and Picture Sequence episodic memory tests and the List Sorting working memory test. Our findings support the appropriateness of using these behavioral tests as traits for separate GWASs. That is, the heritability estimates for the two episodic memory tests matched the ranges found in the literature [42, 74]. All heritability estimates were sufficiently high (ranging from 39.1% for the Penn-Word to 61.7% for List Sorting) to justify a GWAS. Moreover, our results indicate that the three memory tests addressed partially distinct cognitive traits. Although there were significant correlations between the memory test per-SNP effect sizes, explained variances were low. Importantly, this low explained variance was likely not attributable to test unreliability, as indicated by test–retest reliabilities of respectively 0.83 [22], 0.78, and 0.89 for Penn-Word, Picture Sequence, and List Sorting [63]. Together, these observations confirm that the tests indeed effectively measured partially different aspects of memory.

We observed only sporadic overlap in genes across the different memory tests, which is conceptually surprising given that at least the two episodic memory tests were expected to rely on shared biological processes. Nevertheless, this finding is consistent with results of other studies in the field [56]. In fact, we replicated the (near) absence of gene overlap amongst four independent cohorts and between these cohorts and the HCP dataset we used, underscoring the validity of this finding. This lack of overlap on the gene level could be due the fact that memory is a highly polygenic trait, influenced by a large number of genetic variants each contributing very small effects. Detecting such effects requires very large samples, meaning that individual GWAS of memory are easily underpowered. In addition, different memory tasks are likely supported by partly distinct neural circuits, each likely influenced by their own set of genetic variants. This heterogeneity further reduces the likelihood of identifying shared variants across studies, further explaining the observed discrepancy at the gene level. Accordingly, results of the REML analyses indicate a genetic correlation across the different memory types throughout the full genome [5]. However, this shared genetic architecture was not reflected in SNP-level associations with *p* < 1E^−5^, where *p*-values differed notably across memory tests. Thus, the genomic similarity indicated by the GREML results likely resides in low-effect SNPs, suggesting that relying solely on a significance threshold to evaluate genetic similarity between tests may be insufficient. On a gene level, the most convincing finding was the *SERPINE3* gene, which was close to MAGMA genome-wide significance for the Picture Sequence test and is thought to be involved in Alzheimer’s disease and prion diseases [72]. It is of note that we limited eQTL mapping to expression data from brain tissues in order to focus the scope of our biological interpretation. We recognize that ignores potential variants associated through for example gastrointestinal and immune system processes such as those affecting the gut-brain axis [19].

Moving to pathway-level analyses allowed for the joint interpretation of SNPs that individually did not reach genome-wide significance. Using network visualization tools, we identified connections among genes associated with the different memory types. Notably, there was substantial overlap in the pathways identified across both the HCP cohort and other study cohorts. This suggests that while genetic differences may likely exist between memory types, there is convergence on a mechanistic level.

At this mechanistic level, several biological pathways functionally linked to memory were observed. These included the *EPHRIN* neurodevelopmental pathway, which plays a role in hippocampal long-term potentiation [32]. Additionally, pathways such as vesicle transport, membrane trafficking, and protein–protein interactions at synapses were identified, which are essential for axon and synapse development and maintenance [36]. The adaptive immune system, and viral infection pathways also emerged. Recent research increasingly recognizes the impact of the immune system on the nervous system, linking chronic inflammation to several cognitive traits such as memory and executive functioning, as well as neurodegenerative and psychiatric disorders such as major depression disorder and Alzheimer’s disease [31, 41, 61, 66].

Furthermore, our analysis revealed several signaling cascades, including G-protein coupled receptors, WNT signaling, VEGF signaling, and calcium and sodium dependent signaling (*KCNN3*, *SLC4A4*). The latter finding highlights the importance of calcium- and sodium-dependent signal conduction in neurodevelopment and neural processing [71]. Furthermore, general processes such cell cycle, apoptosis, and RNA polymerase II transcription were identified. Dysfunction of these pathways is known to cause severe developmental disorders [38].

The structural variant 8p23.1 was associated through several genes from the Picture Sequence analysis. An 8p23.1 inversion has been associated with neuroticism, depression, well-being, risk tolerance, and risky behavior [4, 49]. In addition, changes in gene expression associated with the inversion have been detected in nervous system tissues [30, 40]. The 2q21.1 structural variant was associated with *FAM168B*, and the brain-specific *ARHGEF4* through the Penn Word analysis. Several deletions of this segment have been associated with developmental delay, attention-deficit hyperactivity disorder, and epilepsy in family and case studies [12, 46].

Structural variants complicate genome-wide analyses and interpretation [50]. These variants can be seen as a single pleiotropic locus, whereas the variants within the structure can be independently associated with distinct traits while showing high linkage amongst those variants [50]. Thus, they require careful statistical fine mapping and further research for more extensive understanding.

The pathway-level findings provide biological context for our genetic results and support the notion that that distinct memory subtypes partially converge on shared molecular mechanisms and biological pathways. However, several limitations of our study warrant consideration. First, while the HCP provides deep phenotyping and high-quality genetic data, its generalizability is limited by demographic characteristics. The cohort is primarily of European ancestry and spans a relatively narrow age range, potentially limiting the extrapolation of our findings to more diverse populations or different life stages. As such, the validity and robustness of our approach remain to be tested in cohorts with broader ancestral and demographic representation. Second, our analyses focused exclusively on common genetic variants, consistent with the constraints and goals of standard GWAS approaches. However, this focus may overlook important contributions from rare variants, multiallelic SNPs, and structural variants [34, 51]. As a follow-up, it would be valuable to investigate the role of rare and structural variants, which may reveal additional genetic influences not captured by common-variant analyses. Third, although we benchmarked our findings against existing GWAS results and conducted pathway-level analyses to assess biological relevance, we did not include direct functional or molecular validation. Future work incorporating transcriptomic, epigenomic, or experimental approaches will be important to further substantiate and interpret the identified associations. Finally, the pathway overlap permutations require further interpretation. We compare our results with permutations from randomly drawn gene lists, whereas our gene lists are not random but generated through association in the genome-wide analyses. In addition, the overlap from the permutations is not evaluated in light of any phenotype through literature by researchers and may thus be biologically spurious.

In conclusion, our findings suggest that the lack of overlapping results from GWASs on memory may be attributed to low statistical power, due to small sample sizes relative to the number of tested SNPs and their expected effect size distributions [39, 73]. However, we cannot exclude the possibility that these memory traits are genetically distinct. Future studies with larger and more harmonized datasets will be necessary to determine whether increased power yields greater convergence across tasks. Lowering the statistical threshold enabled the discovery of variants, genes, and pathways that otherwise would have been overlooked. We advocate for this approach when studying common, complex traits, particularly in deeply phenotyped datasets with modest sample sizes, where traditional GWAS methods may fall short. Our pathway-level analysis revealed connections among the different memory tests and independent cohorts, indicating shared underlying mechanisms. These findings suggest promising biological processes to initiate imaging genetics studies within and outside the HCP dataset, and warrant further investigation in disease cohorts.

## Data Availability

Data is provided within the manuscript or supplementary information files. All scripts used in this pipeline, including annotation, can be found on GitHub: https://github.com/macsbio/HCP_Memory_GWAS. Access to the Human Connectome Project data used within the manuscript can be requested via https://www.humanconnectome.org/.
